# Laser ionization and spectroscopy of Cu in superfluid helium nanodroplets

**DOI:** 10.1016/j.ijms.2013.12.022

**Published:** 2014-05-15

**Authors:** Friedrich Lindebner, Andreas Kautsch, Markus Koch, Wolfgang E. Ernst

**Affiliations:** Institute of Experimental Physics, Graz University of Technology, Petersgasse 16, A-8010 Graz, Austria

**Keywords:** Resonant multiphoton ionization, Helium droplet, Dopant ejection, Relaxation, Copper cluster

## Abstract

Mass and optical spectroscopic methods are used for the analysis of copper (Cu) atoms and clusters doped to helium nanodroplets (He_N_). A two-color resonant two-photon ionization scheme is applied to study the Cu ^2^P${}_{1/2,3/2}^{\circ }\leftarrow^{2}$S_1/2_ ground state transition. The absorption is strongly broadened for Cu atoms submerged inside helium nanodroplets and a comparison with computed literature values is provided. An observed ejection of the dopant from the droplet is triggered upon excitation, populating energetically lower states. The formation of Cu_*n*_ clusters up to Cu_7_ inside helium nanodroplets was observed by means of electron impact ionization mass spectroscopy.

## Highlights

• First experimental investigation of Cu electronic excitations in helium nanodroplets.
• Agreement is found with computed spectra from literature.
• Cu is observed to be solvated inside the droplets and ejected upon excitation.
• Clusters containing up to seven Cu atoms are formed inside the droplets.

## Graphical abstract

**Figure:**
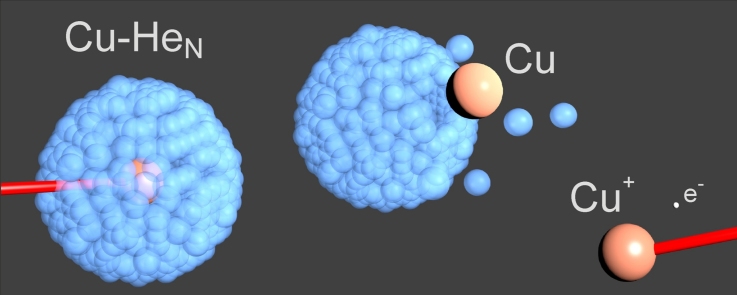

## 1 Introduction

Helium nanodroplets (He_N_) have drawn attention from theoreticians and experimentalists alike, as they offer a confined, transparent, and weakly interacting matrix for a well defined analysis of dopants at low temperatures (0.4 K) [1]. The systematic spectroscopic interrogation of foreign atoms has proven to be a powerful technique for the examination of the dopant properties, as well as a probe for the behavior of the quantum liquid itself [1]. Mass analysis of ionization fragments from atom and cluster doped or pure He_N_, has been widely used to monitor dynamic processes influenced by the superfluid helium environment [1–7].

Besides the very well known technical importance of Cu, applications have been developed in the biosciences where single Cu atom resonance ionization mass spectrometry (RIMS) was used to non-destructively handle and manipulate plant cells that accumulate foreign atoms [8]. Formation of small Cu_*n*_ clusters inside He_N_ will enable the study of high spin states where hardly any data is available but has been shown for alkali and silver dimers and alkali trimers attached to He_N_ [9–12]. One of our goals is the deposition and investigation of structural and magnetic properties of Cu_*n*_ clusters on surfaces as these might differ considerably from those of both their atomic constituents and bulk matter [13,14].

Most experimental and theoretical studies of metal doped He_N_ are concerning alkali atoms. It is well established that alkali atoms reside on the droplet surface [1,9,15,16] because of their large van der Waals radius, while most other atoms are dissolved inside the droplet [5,6,17–19]. Helium can be viewed as a probe to explore the electronic structure of an excited atom [20] and the solvation of atoms inside He_N_ can be extracted from absorption broadenings and line shifts [1,21]. The (*n* − 1)d^10^ *n*s electron configuration of coinage metal atoms can be considered alkali like, so these species are well suited for spectroscopic analysis and were already doped to various matrices. In He droplet experiments, preferably silver was used as a single atom dopant [5,19,22] or to form larger clusters [13,23]. Doping of Cu was reported for the investigation of the bonding between Cu_*n*_ clusters and organic molecules [24], the formation of unusual Cu_*n*_ clusters [14], and Cu was also implanted in a He fountain [25]. Matrix isolated copper was found to undergo nonradiative relaxation and fluorescent emission on forbidden lines [26] where transitions of valence electrons show a strong broadening and blueshift, while inner-shell transitions are practically unshifted [27]. An investigation of the solubility of coinage metals suggests that shell formation around the dopant is expected with the onset of a second shell for more than 25 He atoms and the formation of a compact solvation shell for larger clusters [21].

We were able to measure the absorption of the Cu–He_N_ system to provide comparison to theoretical studies [21,20]. An *ω*_1_ + *ω*_2_ resonance ionization spectroscopy (RIS) scheme is used to study the excitation with one photon utilizing a resonant transition from the ground state and a second photon providing the energy to ionize, a scheme that was also used in free Cu atom spectroscopy [28] for bare atoms. Here we present the results from a mass selective laser spectroscopic study of the first strong ground state excitation of Cu doped He_N_ and mass spectroscopic observation of small Cu_*n*_ clusters formed inside the droplets.

## 2 Experimental

The experimental setup follows the design of a HElium NanoDroplet Isolation (HENDI) spectroscopy apparatus described in detail in previous publications [6,29]. He_N_ are formed by a supersonic expansion of He gas (purity 99.9999%) through a cooled nozzle (closed cycle two-stage cryocooler, *T*_0_ = 13–20 K nozzle temperature, *p*_0_ = 5 MPa stagnation pressure, *d*_0_ = 5μm orifice diameter) into vacuum. The He condenses to form clusters following a log-normal size distribution with, under these conditions, typical maxima in the distribution of ${\hat{N}}_{p_{0},T_{0}}$ = ${\hat{N}}_{5,13\text{–}20}$ = 7200 − 1400 atoms and *r* = 4.3 − 2.5 nm (mean droplet size $\bar{N}$ = 21 200 − 4000). As the spectroscopic linewidth of dopant transitions is partially due to inhomogeneous broadening from the droplet size distribution, the largest intensity within the linewidth has to be assigned to the droplet size represented by the maximum of the log-normal size distribution. For this reason, we prefer to list the maxima rather than the average sizes (see also for details Ref. [30]). The droplets pass a skimmer (*d* = 300 μm) to shape a He_N_ beam traveling toward a pickup oven located in a separately pumped vacuum chamber where the He_N_ statistically pick up one or more Cu atoms. The resistively heated Cu evaporation source consists of a tungsten wire heated alumina (Al_2_O_3_) coated crucible covered with a slitted molybdenum lid with a slit length of 25 mm, arranged parallel below the droplet beam. Gas phase Cu atoms are emitted from the slit and intersect the He_N_ beam at right angles, hence the crossed beam geometric layout – including 5 small apertures to collimate the droplet beam – ensures that no free atoms reach the detection region 1.4 m downstream. For the doping with single Cu atoms, oven temperatures of typically 1000–1100 °C are necessary in this setup. For the formation of Cu_*n*_ clusters inside He_N_, the experimental conditions are chosen to favor the pickup of, on average, more than one Cu atom per droplet, meaning higher crucible temperatures (1100–1300 °C) and large droplets (${\hat{N}}_{5,13.5}\approx$6800 corresponding to an average size of $\bar{N}$ = 18 300). A quadrupole mass spectrometer (QMS, Balzers QMG 422) with counter (Stanford Research SR400) is located at the end of the main vacuum chamber. It can be oriented in two ways to intersect the He_N_ beam with a laser beam either at right angles for photoionization (PI) or antiparallel for beam depletion (BD) measurements. For analysis of the embedded Cu_*n*_ clusters by means of electron impact ionization mass spectroscopy, the QMS is equipped with a crossed beam electron bombardment ion source. Here, the He_N_ beam is chopped for differential counting with a home-built two channel counter.

For the Cu excitation in the UV spectral region, the radiation from an excimer (XeCl, Radiant Dyes RD-EXC-200) pumped dye laser (Lambda Physik FL3002, dye DCM) with ∼25 ns pulse duration and 100 Hz repetition rate is frequency doubled with a KDP crystal (second harmonic generation, SHG). Excitation wavelengths are determined from the fundamental wavelength measured with a wavemeter (Coherent Wavemaster). For Resonant two Photon Ionization (R2PI), part of the excimer laser beam (*λ* = 308 nm) is coupled out of the dye laser pump beam and guided toward the HENDI apparatus simultaneously with the tunable dye laser beam. Both are focused and overlapped inside the ionization region of the QMS (laser fluence: SHG ≈ 7 mJ/cm^2^, XeCl ≈ 5 mJ/cm^2^). The R2PI and BD spectra presented are exclusively recorded at 63 u, the mass of the most abundant Cu isotope. For the observation of photoionized fragments, the mass filter is scanned while ionization is accomplished with fixed laser wavelengths. Overall, this setup allows us to perform resonance ionization spectroscopy of Cu–He_N_ complexes with mass selective ion detection and electron impact ionization mass spectroscopy.

**Fig. 1:**
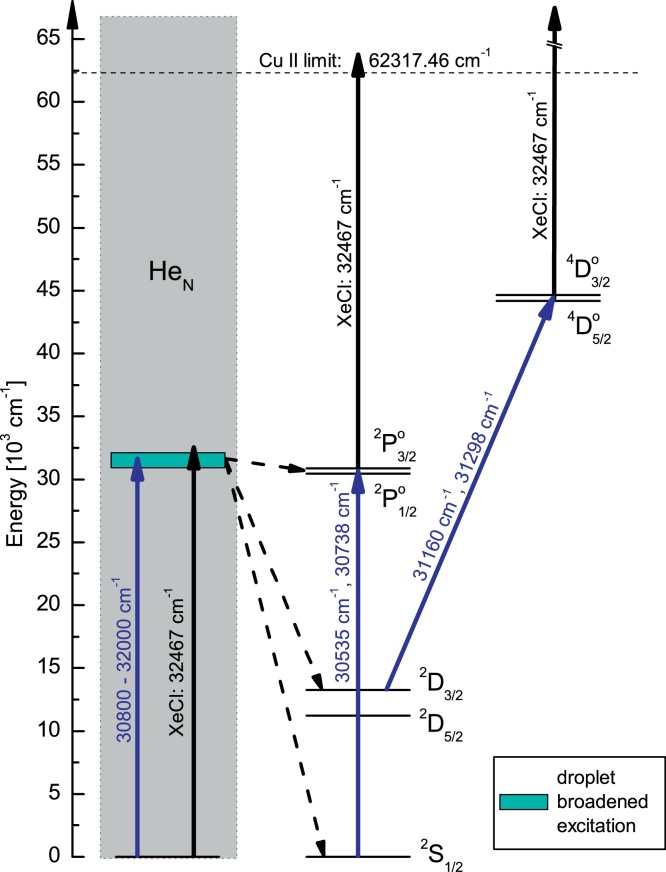
Energy-level diagram of Cu with the observed absorption paths indicated (upwards pointing arrows). The shaded rectangle indicates the excitation broadening due to the He_N_ and the dashed arrows stand for nonradiative relaxation processes. Note that the ^2^P${}_{1/2,3/2}^{\circ }$ and ^4^D${}_{5/2,3/2}^{\circ }$ spin–orbit splitting is not drawn to scale.

The Cu ground state (^2^S_1/2_) electron configuration is [Ar]3d^10^4s. In principle, the ground state absorption measurement follows the simple R2PI excitation scheme (see Fig. 1) of Ref. [28]. For all atoms solvated inside He_N_, the first resonant excitation step is accomplished by the dipole allowed transitions D1 ($\tilde{\nu }$ = 30 535 cm^−1^) and D2 ($\tilde{\nu }$ = 30 783 cm^−1^) [31] to the ^2^P${}_{1/2}^{\circ }$ and ^2^P${}_{3/2}^{\circ }$ (electron configuration: 3d^10^4p) states, respectively. The Cu II limit lies at $\tilde{\nu }$ = 62 317 cm^−1^ which cannot be reached from the Cu^★^ intermediate ^2^P${}_{1/2,3/2}^{\circ }$ states with a photon of $\tilde{\nu }<$ 31 782 cm^−1^ and is thus accomplished by the absorption of a XeCl laser photon ($\tilde{\nu }$ = 32 468 cm^−1^).

## 3 Results and discussion

### 3.1 Resonant two photon ionization of Cu–He_N_

**Fig. 2:**
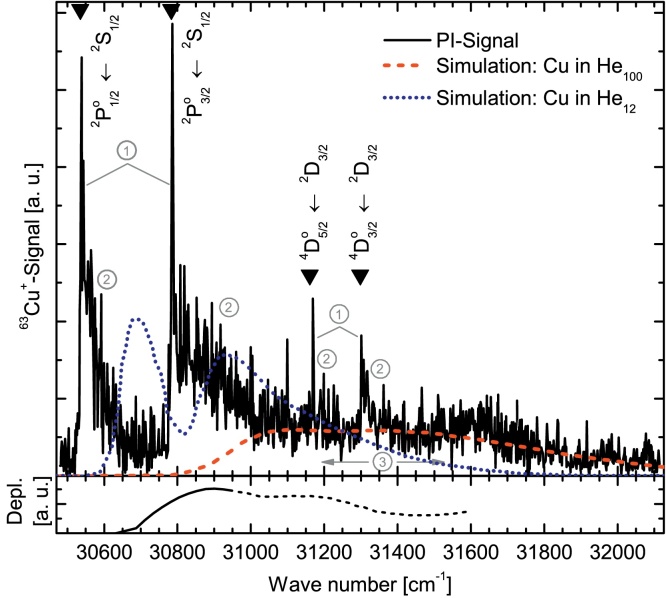
Top: Two-color two-photon ionization spectrum of Cu doped He_N_ (mean radius 3.9 nm) in the energy region of the ^2^P${}_{1/2,3/2}^{\circ }\leftarrow^{2}$S_1/2_ ground state transition. The free atom transition energies are indicated with triangles. Computed absorption spectra for CuHe_100_ (dashed red) and CuHe_12_ (dotted blue) are redrawn from Ref. [21] and scaled for best comparability. Numbers correspond to the features described in the text. Bottom: Beam depletion spectrum of Cu doped He_N_. The dashed signal might not be considered reliable due to large noise. (For interpretation of the references to color in this figure legend, the reader is referred to the web version of the article.)

A number of characteristic absorption features is observed with R2PI, as one laser is scanned from 30 470 cm^−1^ to 32 120 cm^−1^ while the second laser is kept constant at 32 468 cm^−1^. In the upper panel of Fig. 2 the recorded spectrum is plotted together with the computed vertical excitation spectra for CuHe_12_ (dotted blue curve) and CuHe_100_ (dashed red curve) [21] in the ^2^P${}_{1/2,3/2}^{\circ }\leftarrow^{2}$S_1/2_ transition energy region. The ions are detected exclusively at the mass of the most abundant ^63^Cu isotope. Most obviously, it consists of (1) sharp peaks at the catalogued Cu gas phase transition energies which are (2) broadened on their high energy side by up to ∼200 cm^−1^ wide shoulders. These features are discussed below, while first the broad structure (3) stretching over ∼900 cm^−1^ between $\tilde{\nu }$ = 30 900 cm^−1^ and $\tilde{\nu }$ = 31 800 cm^−1^ is treated.

In Fig. 2, the broad band (3) ranging from 30 900 to 31 800 cm^−1^ is assigned as ^2^P${}_{1/2,3/2}^{\circ }\leftarrow^{2}$S_1/2_ excitation of Cu inside the droplet, in reasonable agreement with the calculation of Cargnoni and Mella [21] for CuHe_100_. This is characteristic for outer shell transitions of atoms surrounded by the droplet. Cavities are formed around the dopant and a qualitative understanding of spectral line broadening and shift is provided by the size-dependent energy of the atomic bubble state. During the orbital expansion of a vertical electronic excitation the He atoms inside the cluster cannot readjust their positions [21] so the Pauli repulsion from the surrounding matrix strongly perturbs the dopant valence electron orbitals.

The solvation of metal atoms inside He_N_ is often described using the model by Ancilotto et al. [15] which aims to predict the solvation of a dopant with a dimensionless parameter of *λ*≥ 1.9. The calculated value of *λ* = 2.9 [32] for Cu atoms inside He_N_ and the distinct Cu–He well depth of −28.4 μhartree (−0.77 meV) estimated with ab initio ground state pair potentials [21] is qualitatively supported by our experimental findings. Because of the similar outer shell electron configurations [21,19], similarities between the Cu–He and Ag–He interaction potentials were found [21] which leads to comparable computational and experimental results, apart from the two spin–orbit components that are clearly separated in the case of Ag due to the larger spin–orbit splitting.

The first laser excitation is accomplished while the Cu atom resides inside a droplet with ${\hat{N}}_{5,15}$ = 5200 He atoms (*r* = 3.9 nm). The observed broad and blueshifted absorption (3) agrees well with the simulated absorption for a cluster size of *n* = 100 He atoms [21]. Only the first two solvation shells around the Cu are accounted for to play a role for the excitation shift [21], and further influence for larger cluster sizes is dismissed by the authors. Recent calculations by Mateo et al. [22] revealed that spectra of impurities like Ag, fully solvated inside large enough droplets, are independent of the droplet size and comparable to doped bulk liquid helium. To our knowledge, no experimental data is available for excitation spectra of atomic Cu solvated in bulk liquid He.

Beam depletion measurements (Fig. 2, bottom panel) which are sensitive to the initial ground state single photon absorption of Cu inside He_N_ neglect any photon-induced secondary effects described below. The BD spectrum shall be compared to the computed CuHe_100_ (dashed red curve) vertical excitation spectrum representing the in-droplet excitation best. The onset of the droplet broadened structure lies approximately 100 cm^−1^ to the red of the computed absorption, reaching the maximum absorption level already at 30 800 cm^−1^. So we attribute the deviation from computational results [21] either to saturation effects in the experiment [17] or to the uncertainty in the Lax approximation, that tends to slightly overestimate the energy gaps due to the lack of zero point energy corrections.

While the broad absorption band originating from Cu inside He_N_ can be well assigned, we will now discuss the strongly increased ion yield (1) at and (2) in the vicinity of the free atom ground and excited state transitions. As described above, the experimental setup prevents free atoms from reaching the PI-region from the evaporation source. We hold a photo-induced ejection mechanism responsible for the production of un- and weakly perturbed Cu atoms, like it was predicted by F. Cargnoni and M. Mella in the form of a “spit out” from the droplet following the ^2^P${}_{1/2,3/2}^{\circ }\leftarrow^{2}$S_1/2_ excitation [21]. The spin changing D–D transition (Δ*L* = 0, Δ*S* = 1) should be dipole forbidden but has a low free atom transition strength [31] and as in other doped helium droplet spectroscopy [33], may even become more allowed in the presence of the helium environment. The relatively intense signal from the ^4^D${}_{3/2,5/2}^{\circ }\leftarrow^{2}$D_3/2_ transitions points toward a strong population of the metastable ^2^D_3/2_ state. Exciplexes which form when one or several host atoms (helium) penetrate into the nodal region of the excited valence electron's density distribution [27], are responsible for a fast nonradiative relaxation due to the crossing of the ^2^Σ^+^, ^2^Π_1/2_, ^2^Π_3/2_ and the ^2^Σ^+^, ^2^Π_1/2_, ^2^Π_3/2_, ^2^Δ_3/2_, ^2^Δ_5/2_ potential energy curves of the Cu–He diatomic which converge asymptotically to the atomic ^2^P and ^2^D doublets [20], respectively. The exciplex formation is particularly favored for alkali and coinage metal atoms because of the dumbbell-shaped *nP* orbitals. The elucidated mechanism might also be extended to nonresonant excitation in the vicinity of the resonant state. One excimer laser photon may provide excitation near the resonant transition (see Fig. 1) and lead to ejection and relaxation of a Cu atom. After relaxation, the Cu atoms experience a repeated resonant excitation followed by ionization. Free Cu atoms accompanying the Cu doped He_N_ beam were never observed by us and are not expected due to our “crossed beam” pickup-geometry. The sharp lines (1) at the wavenumbers of the free Cu ^2^P${}_{1/2}^{\circ }\leftarrow^{2}$S_1/2_ and ^2^P${}_{3/2}^{\circ }\leftarrow^{2}$S_1/2_ transitions indicate that within the pulse length of our laser (∼25 ns) a Cu atom can be excited inside the droplet, be ejected from the droplet, relax to the ground state, and be resonantly ionized through the two ^2^P states. Furthermore, the spectrum in Fig. 2 shows spectral bands extending from the free atom transitions toward higher wavenumbers (2). They have a similar shape as the simulated bands by Cargnoni and Mella [21] for Cu in He_12_, which are nevertheless shifted by about 130 cm^−1^ to the blue, about the same amount as the shift for simulated Cu–He_100_ against our depletion band. We conclude that our measured bands (2) derive from small Cu–He_*n*_ (*n* = 1, 2, 3) complexes ejected from the droplet along with the free atoms mentioned above. While our ion mass scans only show (CuHe)^+^ and (CuHe_2_)^+^, recent calculations of Cargnoni et al. [20] predict that the Cu^★^(^2^P) state is capable of binding up to 5 He atoms. Simulations of excitation spectra of the complexes CuHe to CuHe_5_ would shine more light on our observations. The overall agreement between the calculations [21] and our spectra is certainly not perfect but shows the right tendency considering the complexity of the system. The deviation of 100–130 cm^−1^ is similar as in the case of Ag atoms solvated in He_N_ [18,21].

### 3.2 Electron impact ionization of Cu_*n*_He_N_

**Fig. 3:**
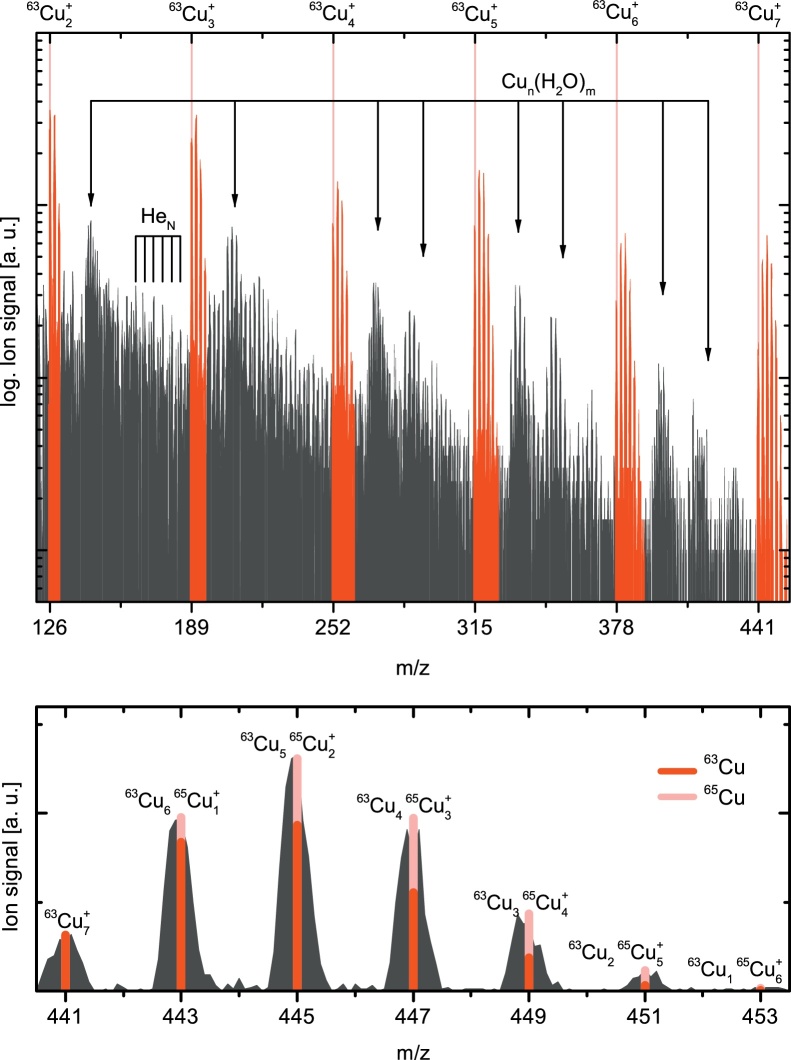
Top figure: Mass spectrum of Cu_*n*_ clusters (red pattern) embedded inside He_N_, detected by electron impact ionization. Bottom figure: Comparison of measured and calculated isotopic composition of the Cu_5_ cluster. (For interpretation of the references to color in this figure legend, the reader is referred to the web version of the article.)

Fig. 3 (upper panel) shows an electron impact ionization mass spectrum of Cu doped He_N_ from Cu_2_^+^ to Cu_7_^+^. The formation of Cu_*n*_ clusters is confirmed by the distinct patterns according to the isotopic composition of ^63^Cu and ^65^Cu. A comparison with the calculated binomial distribution for the Cu_7_^+^ cluster is shown in the lower panel of Fig. 3. Cluster sizes of up to seven Cu atoms were observed, limited by the maximum QMS detection range. The energy introduced by the pickup of seven copper atoms can be estimated according to Lewerenz et al. [34] (discussed in more detail in Ref. [35]) and leads to the evaporation of 2700 ± 100 He atoms, together with the binding energy of the Cu_7_ cluster [36] a total number of 17 000 ± 2000 He atoms are evaporated during the Cu_7_ cluster formation. This is realistic since, according to the log-normal distribution (${\hat{N}}_{5,13.5}\approx 6800$), 38% ± 5% of the droplets are of sufficient size to survive the formation of Cu_7_. Odd-even cluster ion yield oscillations are apparent in the signal integrated over each specific isotopic cluster composition, but are less prominent than observed for Ag [23]. These oscillations can be attributed to the electronic shell structure of the coinage metal clusters, where a cluster with an even number of electrons (odd numbered cluster ion) is more stable than one with an odd number of electrons (even numbered cluster ion) [37]. However, due to various different contributions of droplet size dependent electron impact ionization of Cu_*n*_ inside He_N_, possibly accompanied by fragmentation, we note that the real Cu_*n*_ flux rate cannot easily be deduced from the measured Cu_*n*_^+^ ion yield. Finally, the pickup of water from the residual gas in the vacuum chamber leads to the occurrence of patterns between the cluster masses, originating from Cu_*n*_(H_2_O)_*m*_^+^ compounds and their fragments. An investigation of the electronic properties of such metal–water clusters in the gas phase was recently reported [38]. Further equidistant peaks, separated by 4 u, correspond to the well known He_N_ fragment ions.

Successive doping of the droplet beam with Cr [35] and Cu leads to the formation of mixed metal clusters inside the He_N_. CrCu, CrCu_2_, Cu_2_Cr, and CrCu_3_ were successfully detected by means of electron impact ionization mass spectroscopy. These species are promising candidates for spectroscopic investigation on He_N_, as only little data is available [39] and the mixed clusters may possess interesting magnetic properties [40].

## 4 Conclusions

In this work, we examine the absorption and relaxation mechanisms of Cu atoms embedded inside He_N_ by means of laser ionization spectroscopy with mass selective detection and compare them to computational results from literature [21]. A broad and approximately 700 cm^−1^ blueshifted absorption band is observed as depletion spectrum and is assigned to Cu atoms solvated inside the droplets. Predicted photoinduced ejection was observed including the relaxation to the ground and intermediate ^2^D states together with the formation of small Cu–He_*n*_ clusters. For a better comparison with calculations, the computation of absorption spectra for Cu–He_*n*_ clusters with less than 12 He atoms or for Cu atoms residing on the droplets surface would be helpful.

The successful formation of small Cu_*n*_ clusters and Cu_*n*_(H_2_O)_*m*_^+^ compounds inside He_N_ was shown for up to seven Cu atoms and evidence is provided that a significant fraction of the droplets survive this cluster formation. This will allow systematic spectroscopic studies of electronic spectra of these systems.
